# In Vivo 3D Meibography of the Human Eyelid Using Real Time Imaging Fourier-Domain OCT

**DOI:** 10.1371/journal.pone.0067143

**Published:** 2013-06-21

**Authors:** Ho Sik Hwang, Jun Geun Shin, Byeong Ha Lee, Tae Joong Eom, Choun-Ki Joo

**Affiliations:** 1 Department of Ophthalmology and Visual Science, Seoul St. Mary's Hospital, College of Medicine, Catholic University of Korea, Seoul, Korea; 2 Advanced Photonics Research Institute, Gwangju Institute of Science and Technology, Gwangju, Korea; Zhongshan Ophthalmic Center, China

## Abstract

Recently, we reported obtaining tomograms of meibomian glands from healthy volunteers using commercial anterior segment optical coherence tomography (AS-OCT), which is widely employed in clinics for examination of the anterior segment. However, we could not create 3D images of the meibomian glands, because the commercial OCT does not have a 3D reconstruction function. In this study we report the creation of 3D images of the meibomian glands by reconstructing the tomograms of these glands using high speed Fourier-Domain OCT (FD-OCT) developed in our laboratory. This research was jointly undertaken at the Department of Ophthalmology, Seoul St. Mary's Hospital (Seoul, Korea) and the Advanced Photonics Research Institute of Gwangju Institute of Science and Technology (Gwangju, Korea) with two healthy volunteers and seven patients with meibomian gland dysfunction. A real time imaging FD-OCT system based on a high-speed wavelength swept laser was developed that had a spectral bandwidth of 100 nm at the 1310 nm center wavelength. The axial resolution was 5 µm and the lateral resolution was 13 µm in air. Using this device, the meibomian glands of nine subjects were examined. A series of tomograms from the upper eyelid measuring 5 mm (from left to right, B-scan) × 2 mm (from upper part to lower part, C-scan) were collected. Three-D images of the meibomian glands were then reconstructed using 3D “data visualization, analysis, and modeling software”. Established infrared meibography was also performed for comparison. The 3D images of healthy subjects clearly showed the meibomian glands, which looked similar to bunches of grapes. These results were consistent with previous infrared meibography results. The meibomian glands were parallel to each other, and the saccular acini were clearly visible. Here we report the successful production of 3D images of human meibomian glands by reconstructing tomograms of these glands with high speed FD-OCT.

## Introduction

Dry eye, or keratoconjunctivitis sicca, is defined as “a disorder of the tear film due to tear deficiency or excessive evaporation that causes damage to the interpalpebral ocular surface and is associated with symptoms of discomfort” [1]. An estimated 10 million people in the United States have dry eye syndromes [2]. Dry eye syndrome patients have the following symptoms: foreign body sensation, burning, stinging, itching, dryness, soreness, heaviness of the lids, photophobia, and ocular fatigue. Ocular irritation caused by tear film instability is one of the most common problems encountered by eye care practitioners. Meibomian gland dysfunction (MGD) is one potential cause of dry eye syndrome. Meibography is helpful for diagnosing MGD.

The meibomian glands are modified sebaceous glands in the eyelid that secrete lipids onto the preocular tear film [3]. MGD is thought to lead to reduced lipid secretion, causing excessive evaporation of tears and dry eye syndrome. Methods to photographically document MGD have been introduced that essentially use trans-illumination biomicroscopy [4]–[8]. However, these methods are not widely used, probably because of patient discomfort during the examination, resulting from such factors as glare, heat, and pain induced by direct application of the probe to the eyelid [9]. To overcome such inconvenience, noncontact meibography using infrared light was developed, which is used in research and clinics [6], [9], [10].

Recently, we reported obtaining tomograms of meibomian glands from healthy volunteers using commercial anterior segment optical coherence tomography (AS-OCT) (Visante, Carl Zeiss Meditec, Dublin, CA, USA), which is widely employed in clinics for the anterior segment (including the cornea and anterior chamber angle)[11]. Noncontact meibography using infrared light provides *en face* images of the meibomian glands while OCT meibography supplies tomograms (B-scan) of these glands.

We can create a 3D skull image by reconstruction of the serial axial tomograms of the skull computed tomography (CT). This is a typical example of 3D reconstruction of medical images in hospitals. In the same manner, we could produce a 3D meibomian gland image by reconstruction of serial OCT images of the gland. However, the creation of 3D images was not possible with AS-OCT, because it does not possess a 3D reconstruction function. Thus, in this study, we report the creation of 3D images of meibomian glands by reconstructing the tomograms of these glands using high speed Fourier-Domain OCT (FD-OCT) developed in our laboratory.

## Materials and Methods

This research was carried out at the Department of Ophthalmology, Seoul St. Mary's Hospital (Seoul, Korea) and the Advanced Photonics Research Institute of Gwangju Institute of Science and Technology (Gwangju, Korea) with two healthy volunteers and seven patients with MGD in November, 2011. The study followed the principles of the Declaration of Helsinki, and the Institutional Review Board of Seoul St. Mary's Hospital approved the study. The purpose of this study and methods were explained to the subjects and written consent was obtained from them. The two healthy volunteers were: subject A, a 35 year-old man; subject B, a 30 year-old man. Subjects A and B were initially examined by slit-lamp biomicroscopy to confirm the absence of abnormalities of the eyelid and ocular surface. We used slit-lamp biomicroscopy and noncontact meibography to diagnose MGD. We examined the lid margin for thickening, erythema, hyperkeratinization, vascularization, telangiectasia, notching or orifice capping. We applied pressure to the lid margin to observe the nature of the secretions. Corneal and conjunctival staining was performed with fluorescein or rose Bengal dye. Finally, noncontract meibography was performed and the change in the meibomian gland was scored using the meiboscore system.[9] Using these tests, seven patients (age 31–70 years; four males and three females) were diagnosed with MGD.

### 1. Noncontact infrared meibography

First, we took an infrared photograph of the meibomian gland using the infrared meibography system modified from noncontact infrared meibography [9]. Specifically, an infrared charge-coupled-device (CCD) (XC-EI50, Sony, Tokyo, Japan) was assembled onto the slit lamp (Slit Lamp BQ 900® Haag-Streit, Köniz, Switzerland). A commercial 62 mm diameter infrared transmitting filter (Infrared R72, HOYA, Tokyo, Japan) was attached to the front of the objective lens of the slit lamp and covered with adhesive tape. Only infrared light above 720 nm was transmitted. With the subjects seated in front of the slit lamp, the upper lids were everted to expose the palpebral conjunctiva. The infrared images of the meibomian gland were taken using computer software (MultiCam Studio, Euresys, IL, USA) connected to a CCD camera with a magnification of 10× and 25×.

### 2. FD-OCT system

**Figure 1:**
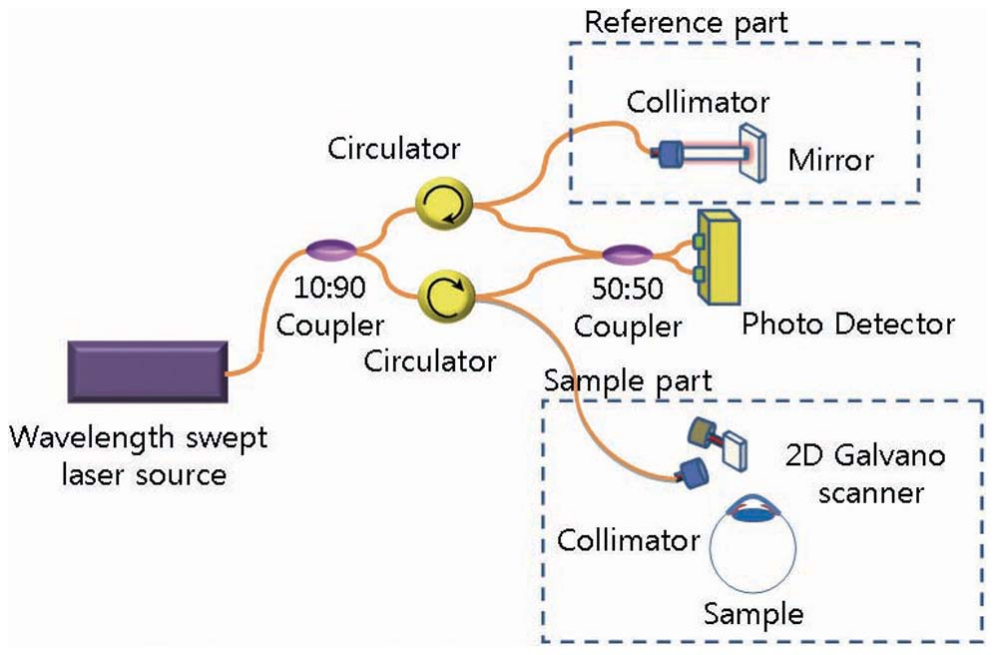
An outline depicting the real time imaging Fourier-Domain OCT (Optical coherence tomography) system based on a high speed wavelength swept laser.

We developed a real time imaging FD-OCT system based on a high-speed wavelength swept laser shown in Figure 1. This high speed wavelength swept laser for the OCT system was built following the Fourier domain mode locking laser (FDML) method [12]. Swept frequency and output power were 52 kHz and 10 mW, respectively. The spectral bandwidth of the swept laser was 100 nm at the 1310 nm center wavelength.

The axial resolution was 5 µm and the lateral resolution was 13 µm in air. Interference signal data from the sample scanner for the human meibomian gland and the reference arm were acquired and transferred to a polarization controller (PC) using a high speed digitizer (PX14400, Signatec, Lockport, IL, USA) with 425 × 10^6^ samples/second and 14 bit resolution. For each A-line, 4096 data points were used to reduce the sensitivity roll-off from the data re-sampling and the interpolation processing. We enhanced the quality of the image by repeating the A-scans twice for each point and averaging them. Then, we performed the B-scan (500 A-scans) and C-scan (200 B-scans). The time required for a 2D image (B-scan, 700×500 pixels) was 21 ms and that for the 3D imaging (C-scan, 700 × 500 × 200 voxels) was 4.2 s (21 ms × 200) irrespective of the subject. A700 pixel height was equivalent to an optical distance of 4.81 mm in air.

### 3. 3D OCT meibography

**Figure 2:**
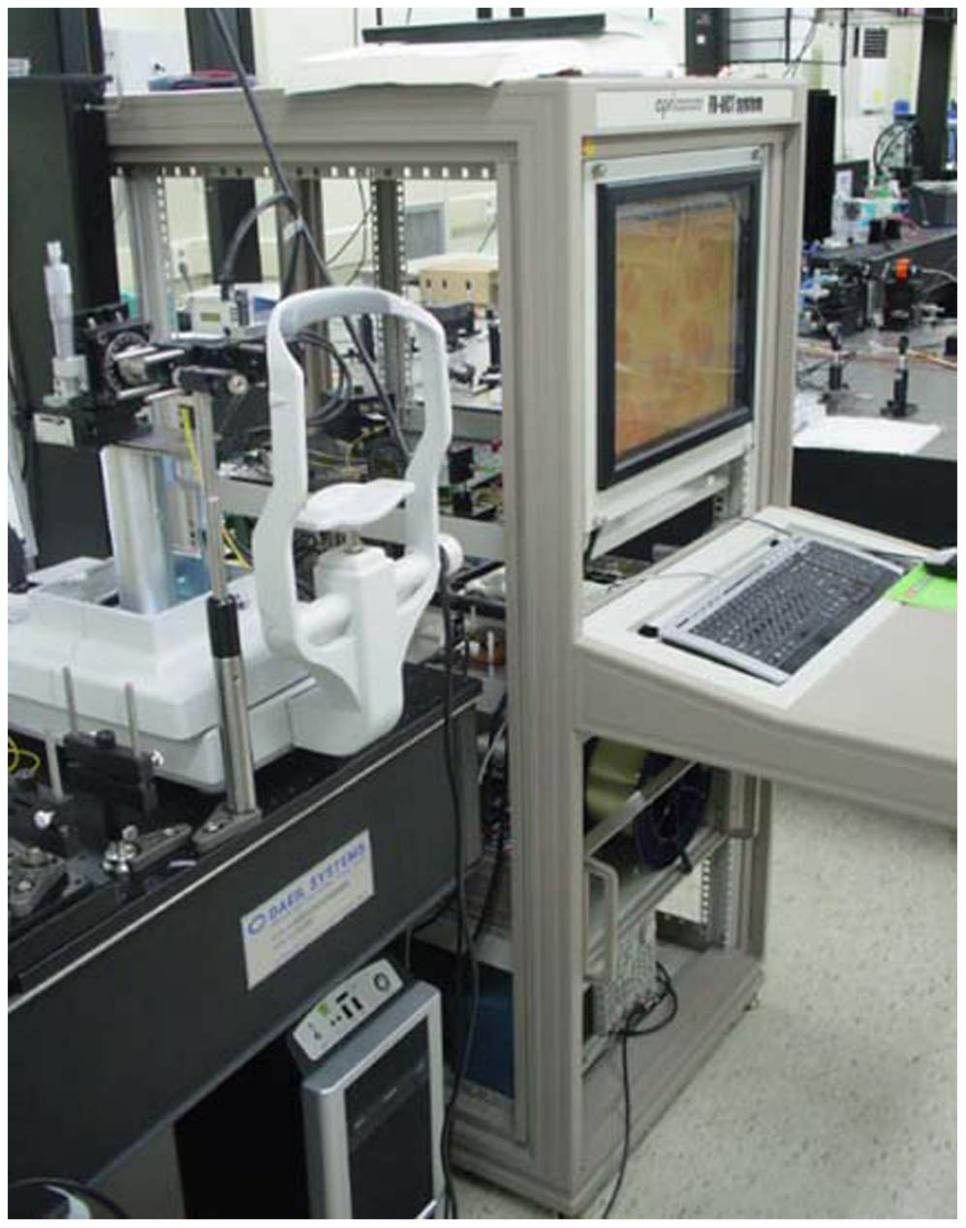
A photograph of the Fourier-Domain OCT (optical coherence tomography) used in this study.

**Figure 3:**
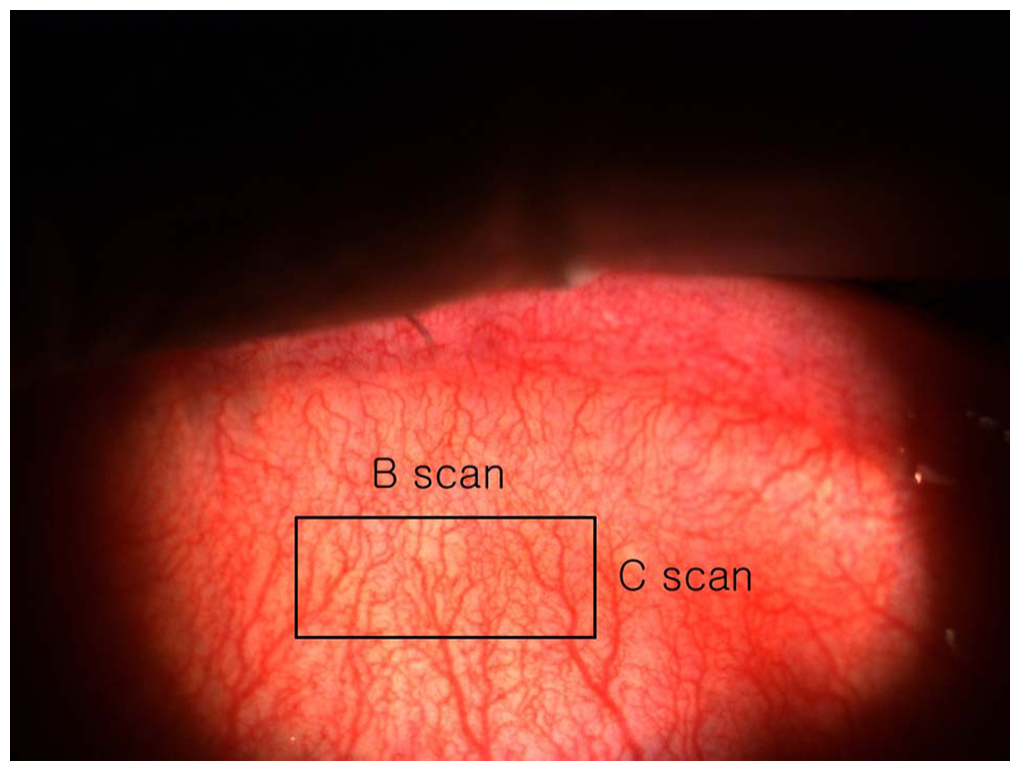
The scan range of the upper palpebral conjunctiva was 5 mm (left to right, B scan (500 A-scans))×2 mm (from upper part to lower part, C-scan (200 B-scans)).

The following procedure was used for the 3D OCT meibography. The subject sat in front of the scanner and placed his or her face on a headrest (Figure 2), and the examiner everted the upper lid. To take a tomogram of the middle of the everted upper lid, the height of the headrest was adjusted. The upper palpebral conjunctiva was scanned, producing tomograms of the meibomian glands using the FD-OCT system. The scan range of the upper palpebral conjunctiva was 5 mm (left to right, B-scan (500 A-scans)) × 2 mm (from upper part to lower part, C-scan (200 B-scans)) (Figure 3).

After completing FD-OCT scanning, the 3D image of the meibomian glands was reconstructed using 3D “data visualization, analysis, and modeling software” (AMIRA software; Mercury Computer Systems, Chelmsford, MA, USA). We cropped the volume data to remove the image of the palpebral conjunctiva and to highlight the region of the meibomian glands. We applied the following image processing protocol to distinguish between the meibomian glands and the conjunctiva structures in the acquired OCT images. First, we found the air-tissue boundary and removed the image of the air region. Second, we flattened each A-line signal of the OCT images to obtain the same relative height of the surface image. Finally, the image of the conjunctiva layer was removed with the same thickness for each OCT image and only the gland images were reconstructed. This image processing was carried out using a vision program developed based on MATLAB 7.1 (MathWorks, Natick, MA, USA).

## Results

**Figure 4:**
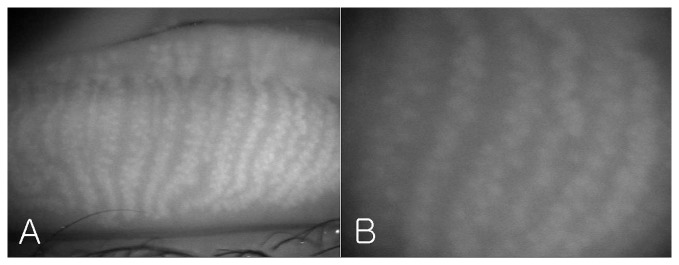
The meibomian glands of the left upper lid of the subject A by modified infrared Meibography (A: ×10; B: ×25).

Figure 4 shows the meibomian glands of the left upper lid of subject A photographed by modified infrared meibography. Approximately 21 well-developed meibomian glands were arranged perpendicular to the lid margin. They displayed typical grape-like patterns and the saccular acini were clearly visible.

**Figure 5:**
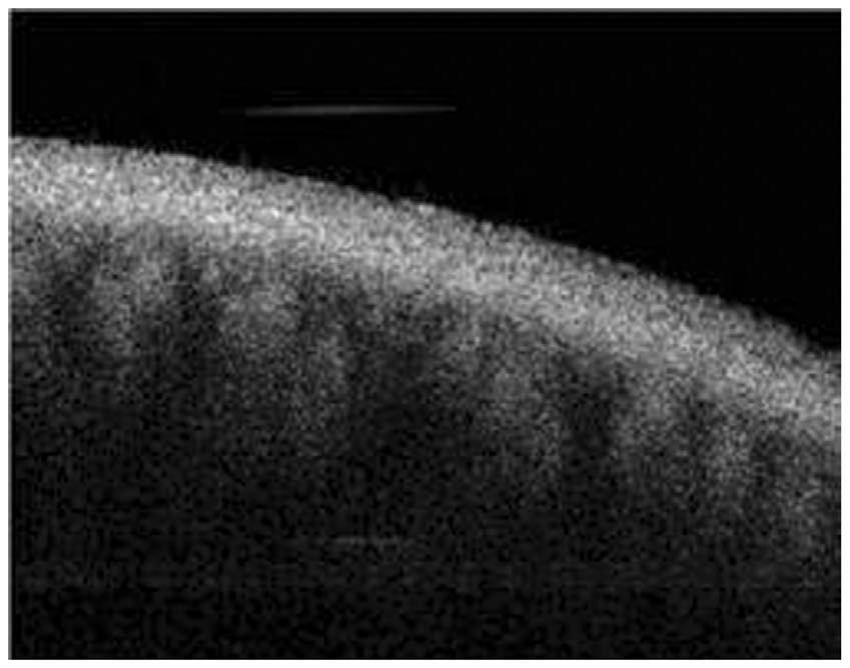
The tomogram (B-scan, 5 mm) of the meibomian glands of subject A by Fourier-Domain OCT (Optical Coherent Tomography).

**Figure 6:**
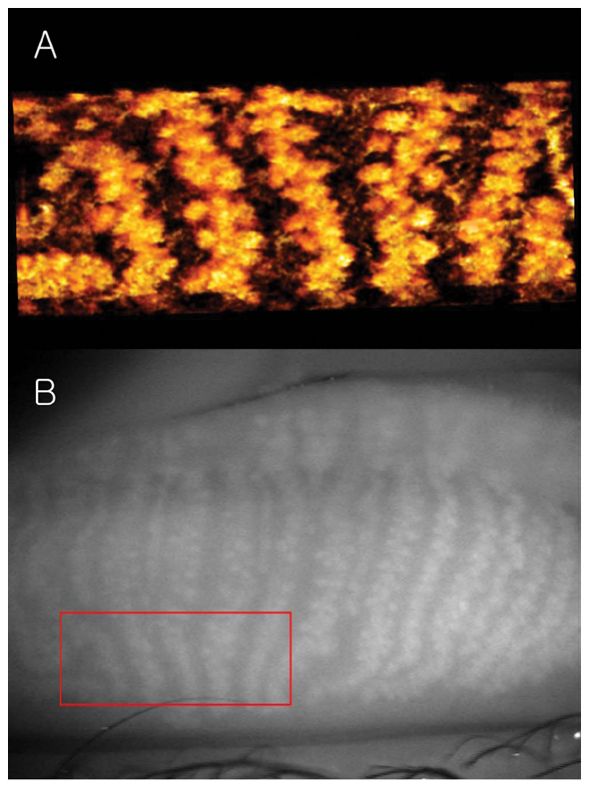
The 3D meibomian gland of healthy subject A (35 years old, male). A: The spherical acini attached to a stalk were clearly visible in the 3D image; B: The 3D meibomian gland image was consistent with the meibomian glands in the rectangle in the infrared images.

**Figure 7:**
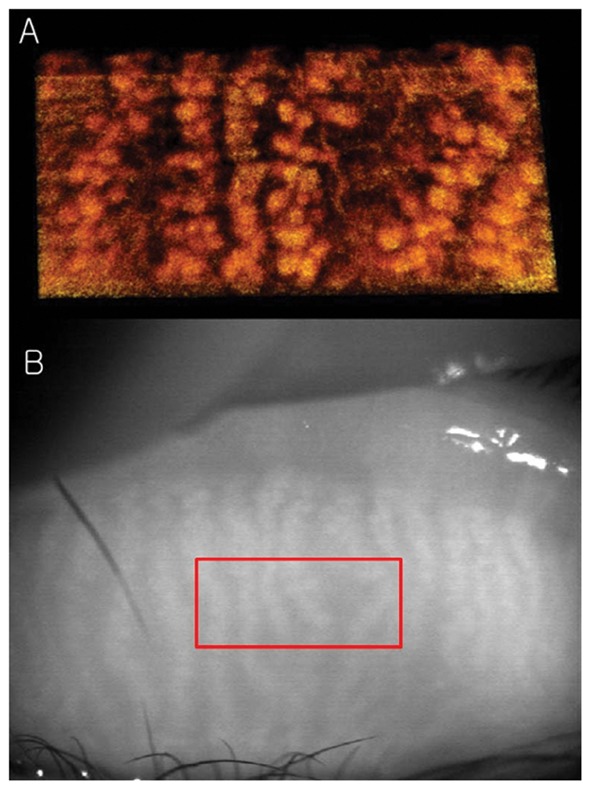
The 3D meibomian glands of healthy subject B (30 years old, male). A: There were extensive networks of branching structures between parallel groups of meibomian glands; B: We could not find the networks in the infrared images of meibomian gland.

**Figure 8:**
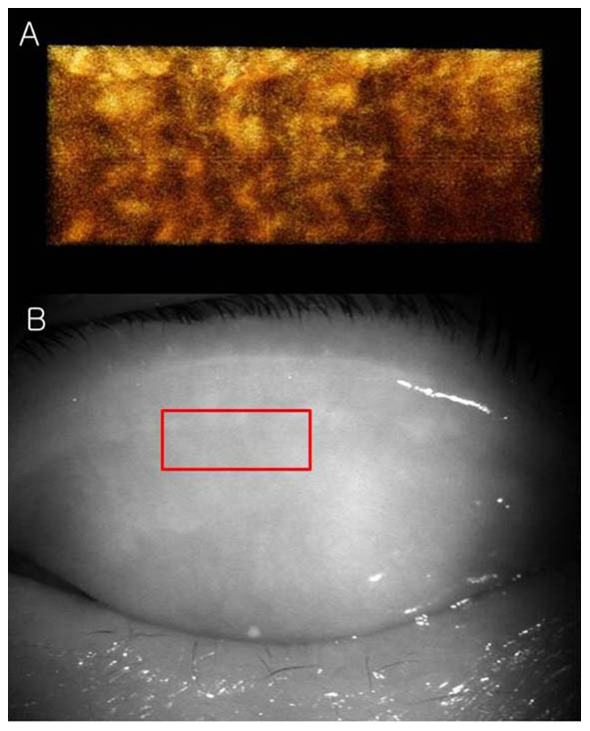
The 3D meibomian gland of subject C (67 years old, male) with severe MGD (meiboscore grade 3). A: The 3D images showed a few meibomian glands with a normal grape-like pattern. We could not find definite acini attached to the central ducts. B: These findings are consistent with the noncontact infrared meibography, which showed nearly complete loss of the meibomian glands.

**Figure 9:**
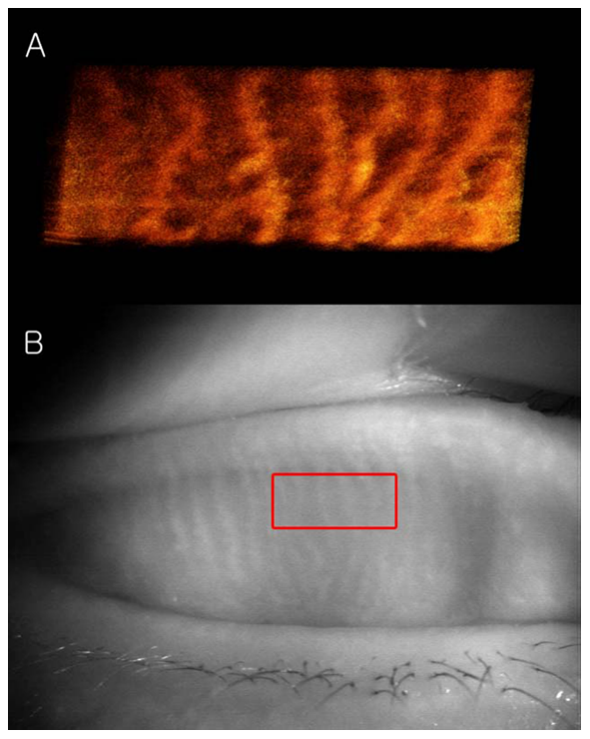
The 3D meibomian gland of subject D (68 years old, female) with mild MGD (meiboscore grade 0 or 1). A: Although the extent of the meibomian gland loss seemed minimal, we saw definite atrophic changes in the acini attached to the central ducts. The contours of the acini were ill-defined and the acini small; B: These findings are consistent with noncontact meibography showing ill-defined acini.

**Figure 10:**
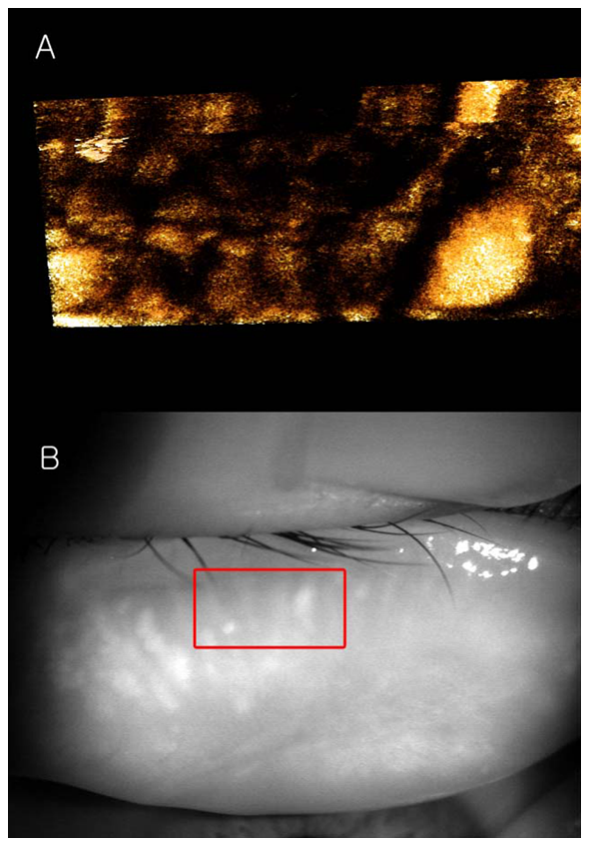
The 3D meibomian gland of subject E (a 31-year-old male) with severe MGD (meiboscore grade 3) and graft-*versus*-host disease. A: We found few meibomian glands with normal morphology, but observed spindle-shaped or globular structures instead; B: The 3D meibomian gland image was consistent with the meibomian glands in the rectangle in the infrared image.

The tomogram (B-scan, 5 mm) of the meibomian glands of subject A by FD-OCT is shown in Figure 5. Many meibomian glands were identified just beneath the palpebral conjunctiva in the OCT image. Figure 6A is a 3D image created by reconstructing 200 images taken by repeating the B-scan of the meibomian glands from the upper part to the lower part (5 × 2 mm) (Figure 6B). The 3D meibomian gland image was consistent with the meibomian glands in the rectangle in the infrared images (Figure 6B). The spherical acini of the meibomian glands were clearer in the 3D image than in the infrared images. A number of acini were attached to a stalk and formed a meibomian gland. Figure 7 shows the 3D meibomian gland of subject B. In this subject, there were extensive networks of branching structures between parallel groups of meibomian glands (Figure 7A). But, we could not find the networks in the infrared image of meibomian gland (Figure 7B). Figures 8 to 10 show the 3D OCT meibography and noncontact meibography of seven MGD patients. Figure 8 shows the 3D meibomian gland of subject C (a 67-year-old male) with severe MGD (meiboscore grade 3). The secretion was turbid when applying pressure to the lid margin and the ocular surface stain was grade 1 according to Oxford scheme [13]; however, we did not find capping of the orifice or notching of the lid margin. The 3D images showed a few meibomian glands with a normal grape-like pattern (Figure 8A). We could not find definite acini attached to the central ducts. These findings are consistent with the noncontact infrared meibography, which showed nearly complete loss of the meibomian glands (Figure 8B). Figure 9 shows the 3D meibomian gland of subject D (a 68-year-old female) with mild MGD (meiboscore grade 0 or 1). Although the extent of the meibomian gland loss seemed minimal, we saw definite atrophic changes in the acini attached to the central ducts. The contours of the acini were ill-defined and the acini small (Figure 9A). These findings are consistent with noncontact meibography showing ill-defined acini (Figure 9B). Figure 10 shows the 3D meibomian gland of subject E (a 31-year-old male) with severe MGD (meiboscore grade 3) and graft-*versus*-host disease. We found few meibomian glands with normal morphology, but observed spindle-shaped or globular structures instead (Figure 10A). These findings are consistent with noncontact meibography showing dropout, shortening, and dilatation of the glands (Figure 10B). The 3D morphology of the meibomian glands of the seven patients with MGD is summarized in Table 1.

**Table 1:** The findings in 3D morphology of meibomian gland of the seven patients with meibomian gland dysfunction.

Patient	Sex	Age	Grading*	Findings				Type
				Drop -out	Shortening	Distortion	Dilation	
C	Male	67	3	o	o			Obstructive
D	Female	68	0–1	o	o			Seborheic
E	Male	31	3	o	o		o	Obstructive
F	Male	50	2	o	o			Seborheic
G	Female	70	1	o	o	o		Seborheic
H	Male	56	3	o	o	o	o	Obstructive
I	Female	53	1	o	o	o		Seborheic

* Meiboscore system by Arita et al[9].

The thickness of the removed layer was approximately 345 µm (50 pixel height for Figure 6A) and 380 µm (55 pixel height for Figure 7A). If we consider the refractive index of human skin tissue as 1.36 at the 1300 nm wavelength range, the meibomian gland appeared from 265 µm and 292 µm under the palpebral conjunctiva surface, respectively [14]. The thickness of the gland structure can be taken as approximately 570 µm for subject A (120 pixel height for Figure 6A) and 428 µm for subject B (90 pixel height for Figure 7A) if we consider the refractive index of the meibomian gland as 1.45 at the near infrared range [15]. The scattering signals were relatively different in the two volunteers. This caused the color difference between Figures 6 and 7. Videos S1 and S2 are movies showing the 3D dimensions of the meibomian glands of subject A using our FD-OCT system.

## Discussion

This is the first study in which *in vivo* 3D images of human meibomian glands were created by reconstructing tomograms of these glands using a high speed FD-OCT with a 1310 nm FDML swept laser.

Recently, Bizheva *et al*. presented tomograms of human meibomian glands using ultra-high resolution OCT. A high-speed, 1060-nm ultra-high resolution OCT system was developed for *in vivo* imaging of the human anterior and posterior eye [16]. The 2D and 3D tomograms were acquired *in vivo* from the everted eyelids of three human subjects. The 2D tomograms showing ‘ductules’ and ‘acini’ were similar to our OCT images (Figure 5), whereas the *en face* images of the 3D tomograms were quite different from our infrared (Figure 4) and 3D images (Figures 6 and 7) of meibomian glands. Their ‘duct-like’ structures of *en face* images have a branching morphology more like vessels than meibomian gland ductules. In our research, the *en face* images are consistent with the grape-like patterns in the infrared images. Therefore, our 3D images were of meibomian glands and not of the other tissues, such as subconjunctival vessels.

Recently, Matsumoto *et al*. reported *in vivo* tomograms of meibomian gland imaging using a confocal microscope (Heidelberg Retina Tomograph II- Rostock Cornea Module, Heidelberg Engineering, Dossenheim, Germany) [17]. If serial tomograms can be used to reconstruct 3D images, high-magnification 3D meibography can be obtained. Whether the software can support this, or if it is possible to overcome involuntary lid movement in such a high-magnification state to obtain satisfactory 3D images, remains uncertain.

We were able to successfully create a 3D image of the meibomian gland using our FD-OCT in this study because of its two major features. First, it is a high speed OCT, which was possible because it is a Fourier-Domain type OCT and uses a high-speed wavelength swept laser. Unlike corneas, everted eyelids can cause motion artifacts due to eyelid movement by the subject or by the finger of the examiner. The A-scan speed of the OCT used in this study was 52 kHz, which is 26 times faster than that of the commercial AS-OCT. It took only 4.2 seconds to scan a 5 × 2 mm area of the upper lid. It was impossible to take 200 tomograms for a 3D image without motion artifacts using the previous AS-OCT, which would require over 166.4 seconds. Second, it uses a light source with a central wavelength of 1310 nm. Because infrared light of 1310 nm can penetrate deeply into tissues, good images of the meibomian glands under the conjunctiva were obtained.

However, some problems remain. First, although the FD-OCT is 26 times faster than the previous AS-OCT, it is still slow in scanning the everted eyelid. During the 4.2 seconds necessary for scanning, minimizing the motion arising from the subject or the examiner is difficult. During examination, everted upper lids have forward-backward, up-down, and left-right movements. At post-processing, forward-backward movement could be partially corrected by the alignment of conjunctival surfaces in the tomograms (B-scans) with each other. However, up-down and left-right movement could not be corrected because there was no reference available. Some commercial OCT instruments have the capability to stabilize during ocular imaging. The OCT system incorporates an aiming target, which the subjects can look at during imaging. However, in 3D OCT meibography, the subject cannot look at the target because of everted upper lids. Furthermore, commercial OCTs have an auto-tracking function, which stabilizes images. In contrast, our system lacks auto-tracking. Addition of a tracking function would greatly reduce motion artifacts.

Second, the resolution of the 3D images remains low. Although the 3D images show more detailed structures than infrared meibography, the contrast is insufficient to identify the gland boundary. To enhance the image resolution, the optimal wavelength with minimum scattering at the conjunctiva should be determined; additionally, attempt other optical methods should be attempted.

Third, we must determine the potential clinical applications of 3D images of the meibomian glands. This OCT system shows the 3D morphology of the meibomian glands in detail. It might be possible to determine the type of MGD (obstructive or hyposecretory) using 3D images. Using noncontact meibography, we can devise a more detailed grading system for MGD than the meiboscore grade.[9] The meiboscore was scored according to loss in the area of the meibomian gland: grade 0, no loss of the meibomian glands; grade 1, area loss less than one third of the total meibomian gland area; grade 2, area loss between one and two thirds; grade 3, area loss exceeded two thirds. Although patient D appeared normal using the meiboscore grading system (grade 0 or 1), 3D OCT meibography shows definite atrophic changes in the acini attached to the central ducts (Figure 9). This imaging is especially helpful for early MGD, such as seen in patient D.

Similar to previous reports, the obstructive MGD group had a significantly higher average meiboscore than the control group, and various meibomian gland changes, such as dropout, shortening, distortion, and gland dilation, were seen in all patients in the obstructive MGD group.[18] The mean meiboscore was significantly lower in the seborrheic MGD group than in the control group.[19] In the seborrheic group, meibomian gland changes such as dropout, shortening, dilation, and distortion were observed and appeared to be within the range of normal age-related changes.

Using the 3D images, we need to develop indices (*e*.*g*., acinus diameter, acinus volume, central duct diameter, meibomian gland volume, volume percentage of meibomian glands in the tarsal plate) of the degree of development of the meibomian glands. For example, quantifying the volume of a meibomian gland structure [20] would allow clinical application of 3D images of the meibomian glands for diagnosing dry eye or MGD. We will apply 3D OCT meibography to a large series of MGD patients and compare with normal subjects.

Fourth, the imaging field (5 × 2 mm) of this system is small compared to the size of the upper eyelids. Meibomian glands loss is often uneven across the eyelid. The scanned area might not be sufficiently large to view lost glands. The 3D morphology of the meibomian glands in the imaging field is not always representative of all of the meibomian glands in the eyelid. In patients with moderate MGD, the glands in the imaging field might even look relatively healthy. Therefore, the positioning of the field appears critical and might produce misleading results. Thus this technique would be useful for MGD only if used in conjunction with noncontact infrared meibography.

We needed two examiners to perform 3D OCT meibography. One examiner held the subject's everted upper lid and the other scans the palpebral conjunctiva using the OCT. The image quality of 3D OCT meibography varies depending on the examiner who held the upper lids (inter-examiner repeatability). The larger the involuntary hand tremor of the examiner, the greater the motion artifact. Even for the same examiner, the OCT meibography varied depending on the position of the imaging field or hand tremor (intra-examiner repeatability).

In this study, we performed 3D OCT meibography of the upper lids. Imaging of the lower eyelids is also possible. However, since the subjects blink their eyes frequently, the motion artifact with the lower eyelids is much greater than that of the upper lids in our experience. In addition, the meibomian glands of the lower lids are shorter than those of the upper eyelids. Therefore, it was sometimes difficult to find the meibomian glands in the small scan field.

In subject B, we found extensive networks of branching structures between parallel groups of meibomian glands (Figure 7). However, we could not identify them. A single meibomian gland is composed of clusters of secretory acini that are arranged circularly around a long central duct and are connected to it by short ductules. The individual glands are arranged in parallel in a single row throughout the length of the tarsal plates in the upper and lower lids [21]. To our knowledge, no reports on collateral arrangements of meibomian glands have been published. Instead, the meibomian glands of the human have a dense meshwork of unmyelinated nerve fibers (nerve plexus) around the acini [22]–[24]. Additionally, it was known that many capillaries are located around the meibomian glands [21]. We believe the ‘network’ between meibomian glands may be a nerve plexus or a capillary, rather than a collateral arrangement of the meibomian glands.

A tomogram of the meibomian glands using OCT (Figure 5) provides information on depth, unlike infrared imaging. Infrared meibography (Figure 4) appears to show the meibomian glands in greater detail. However, it is an *en face* image, lacking depth information. By reconstructing the serial tomograms, we could show the 3D morphology of the meibomian gland. We identified a ‘network’ (nerve plexus of capillaries) between or behind the meibomian glands using 3D OCT meibography. Furthermore, the resolution of 3D meibography is greater than that of infrared meibography.

## Conclusions

We successfully produced 3D images of human meibomian glands by reconstructing tomograms of these glands with high speed Fourier-Domain OCT developed in our laboratory. We believe that this imaging technique can be applied to research on meibomian glands.

## Supporting Information

Video S1 **Three-axis OCT (Optical Coherent Tomography) tomograms of the meibomian glands of subject A.**
(WMV)

Video S2 **The reconstructed 3D volume image of the meibomian glands of subject A.**
(MPG)
